# Collet-Sicard syndrome as an initial presentation of prostate cancer: a case report

**DOI:** 10.1186/1752-1947-5-315

**Published:** 2011-07-14

**Authors:** Rosa Villatoro, Carlos Romero, Antonio Rueda

**Affiliations:** 1 Unidad Oncologia Médica, Autovia A-7, km 187, Hospital Costa del Sol, Marbella, 29603, Spain; 2 Servicio Medicina Interna, Autovia A-7, Km 187, Hospital Costa del Sol, Marbella, 29603, Spain

## Abstract

**Background:**

Collet-Sicard syndrome is caused by lesions at the base of the skull affecting the lower cranial nerves. It is associated with various etiologies of tumoral and other origin. Although this syndrome has been reported previously in the literature, most cases are diagnosed as part of primary disease follow-up. This case is unusual because of the diagnosis of bone metastasis secondary to prostate cancer.

**Case presentation:**

We present the case of a 70-year-old Caucasian man with a three-week history of headache and maxillary pain on the right side together with paresis of the low cranial nerves. This study was carried out with a computed tomography (CT) scan of the larynx and neck and MRI, which revealed a bone lesion at the base of the skull affecting the right occipital condyle and part of the right side of the basilar bone. On the basis of differential diagnosis, a fibrous dysplasia, Paget's disease or metastasis was considered. Finally, and after other studies were performed, a diagnosis of bone metastasis secondary to prostate cancer was established.

**Conclusions:**

We think that this case is curious because it involved an initial presentation of metastatic prostate cancer. It is important this should be considered in the differential diagnosis when a patient with unusual clinical findings is first seen in view of the fact that first-line hormonal treatment may control the disease for months or years.

## Background

Collet-Sicard syndrome is caused by lesions at the base of the skull affecting the lower cranial nerves, which produces dysphonia, displacement of the palate, and atony of the trapezius muscle and sternocleidomastoid, as well as anesthesia of the larynx, pharynx and soft palate. It is associated with various etiologies of tumoral and other origins. The differential diagnosis is important. Among the non-tumoral factors causing Collet-Sicard syndrome, the most common are traumatic events (fractures at the base of the skull, aneurisms, and so on), inflammatory processes (osteomyelitis, Paget's disease, and so on) or other alterations such as diabetes mellitus or porphyrias [1]. However, considering a potential tumor cause in the differential diagnosis is important.

Collet-Sicard syndrome may be diagnosed based on clinical history, a physical examination or imaging studies such as computed tomography (CT) and MRI scans [1]. The site most frequently affected is the petrous apex, although the external auditory canal, the middle ear and the mastoid apophysis can also be involved [2]. The symptoms vary depending on the location of occurrence, producing effects ranging from loss of hearing to tinnitus or disorders of cranial nerve VIII, the jugular foramen or the anterior condylar canal. The latter is the site described in our patient's case [3].

## Case presentation

**Figure 1:**
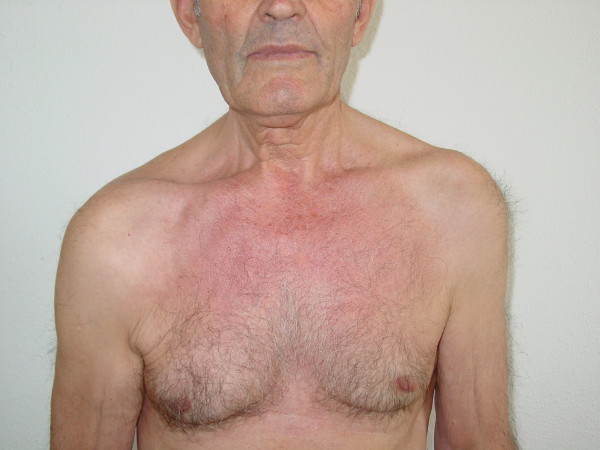
**Paresis of cranial nerve XI**.

**Figure 2:**
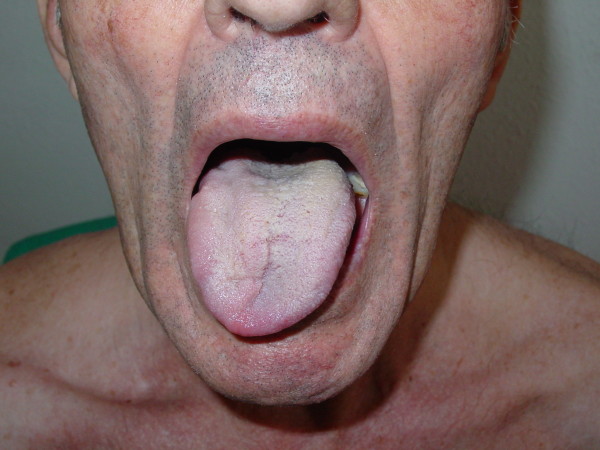
**Paresis of cranial nerve XII**.

We report the case of a 70-year-old Caucasian man, with no significant clinical background, who presented to our casualty department with a three-week history of headache and maxillary pain on the right side, together with the recent appearance of dysphonia and dysphagia for solids. No urinary disorder was reported. A neurological examination revealed a paresis of cranial nerves IX and X, manifested by the displacement of the soft palate to the right and difficulty swallowing. Paresis of cranial nerves XI and XII was also observed, indicated by the lowering of the right shoulder and hypotonia of the right trapezius muscle, and was accompanied by displacement of the tongue toward the right, right-side hypotonia and muscle twitching (Figures 1 and 2). Results of the rest of the physical examination were normal.

**Figure 3:**
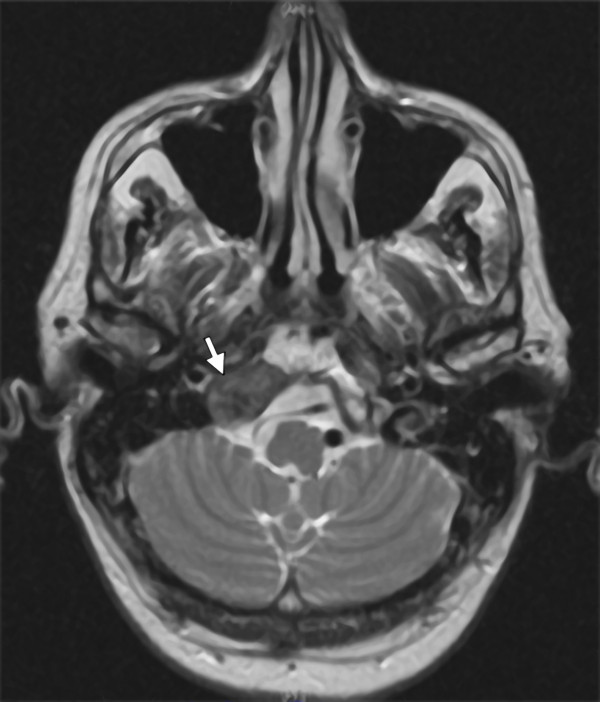
**MRI scan with moderate degree of enhancement at the base of the skull, affecting the right occipital condyle and part of the right side of the basilar bone**.

Blood analysis results revealed an alkaline phosphatase level of 350 UI/L (normal range 44 to 147 UI/L) but no other significant alterations. In view of the paresis of the four lower cranial nerves, a CT scan of the larynx and neck was performed; the CT scan revealed an asymmetrical union between the clivus and the right occipital condyle, adjacent to the jugular foramen, with increased ground-glass bone density. There was no visible lesion to the bone cortex or soft tissues. This study was complemented with an MRI scan, which revealed a bone lesion producing a hypointense signal at sequences T1 and T2. Administration of a gadolinium contrast agent produced a moderate degree of enhancement at the base of the skull, affecting the right occipital condyle and part of the right side of the basilar bone (Figure 3). The image corresponded to a moderately space-occupying blastic lesion, visible in the CT scan, which slightly decreased the caliber of the jugular foramen and the condylar canal. Therefore, fibrous dysplasia, Paget's disease and metastasis were considered in the differential diagnosis.

Subsequently, an additional radiographic examination of the lumbar column and pelvis was carried out; this examination did not reveal any lesions suggestive of Paget's disease. A bone gammagraphy was then requested, and images showed multiple pathological foci of tracer uptake in the right maxilla, the rib cage, right scapula, spine and pelvis. These foci were compatible with disseminated bone metastases. The blood analysis was repeated, and tumor markers were studied. The initial prostate-specific antigen (PSA) value was 21.30 ng/mL.

A physical examination revealed an enlarged prostate with a hard consistency, destructured in the left lobe. Because there was a strong suspicion of prostate neoplasm, a biopsy was performed. The anatomic pathology findings were bilateral common adenocarcinoma, with a Gleason grade of 8 (4+4), affecting 60% of the tissue. There was no presence in the periprostatic adipose tissue and no perineural infiltration.

Following the diagnosis of stage IV prostate adenocarcinoma by metastatic bone dissemination with Collet-Sicard or jugular foramen syndrome, hormone treatment was begun with an antiandrogen. Then, 15 days later, a luteinizing hormone-releasing hormone (LHRH) analog was added and a monthly dose of zoledronic acid was subsequently included. The PSA value during the diagnostic process, prior to the start of antiandrogen therapy, was 71.9 ng/mL.

After three months of treatment, our patient was able to swallow normally, but the dysphonia remained. The rightward displacement of the palate, the lowering of the right shoulder and the atony of the right side of the tongue (paresis of cranial nerves XI and XII) remained unaltered. The latest PSA value was 0.11 ng/mL.

## Discussion

The clinical presentation of metastasis to the temporal bone is uncommon, and few cases have been reported. Nevertheless, its incidence is probably greater than commonly estimated because of the number of cases that remain undiagnosed. The multi-symptom nature of metastatic bone disease tends to produce more incapacitating symptoms than those associated with diseases of the temporal bone.

Various retrospective series of patients presenting with this syndrome have been reported in the literature. Vázquez *et al*. described 21 cases, of which 71% were secondary to neoplasia (57% from paraganglioma and 14% by the direct extension of carcinoma of the cavum) [1]. Imamura *et al*. reviewed the potential mechanisms responsible for metastatic dissemination to the temporal bone. Of the six patients studied, three cases presented hematogenous dissemination (hepatocellular carcinoma, non-microcytic lung cancer and adenocarcinoma of unknown origin), two cases were the consequence of direct invasion by carcinoma of the head and neck, and one case was caused by leptomeningeal carcinomatosis (carcinoma of transitional cell carcinoma of the renal pelvis) [4]. Gloria-Cruz *et al*. selected 212 corpses of patients with non-disseminated neoplasias for an autopsical study of the temporal bone. These authors identified 47 patients with metastasis in the temporal bone, and the involvement was bilateral in 62% of these cases. The most frequently occurring site was the petrous apex, and the hematogenous pathway was the normal route of dissemination [5,6].

The management of Collet-Sicard syndrome consists of treating the cause that originates. In this case, therapy over primary tumor, followed by other measures such as the use of steroids or radiotherapy to help reduce edema and, thus, alleviate symptoms that can be limiting for the patient [7].

## Conclusions

The medical literature contains various descriptions of patients with disseminated prostate cancer who presented with Collet-Sicard syndrome; however, in almost every case, this diagnosis was already known when neurological symptoms began [7-12]. Apart from our patient, only one other case has been reported where metastasis to the temporal bone was the first recognized symptom of the disease [12]. It is important to consider the possibility of the existence of prostate cancer when a patient with an unusual clinical presentation is first seen, in view of the fact that first-line hormonal treatment may control the disease for months or years.

## Consent

Written informed consent was obtained from the patient for publication of this case report and any accompanying images. A copy of the written consent is available for review by the Editor-in-Chief of this journal.

## Competing interests

The authors declare that they have no competing interests.

## Authors' contributions

CR made substantial contributions to the design, and the acquisition and interpretation of data. AR revised the manuscript critically for important intellectual content. RV was a major contributor in writing the manuscript. All authors read and approved the final manuscript.
